# Combination of the PI3K inhibitor Idelalisib with the conventional cytostatics cytarabine and dexamethasone leads to changes in pathway activation that induce anti-proliferative effects in B lymphoblastic leukaemia cell lines

**DOI:** 10.1186/s12935-020-01431-4

**Published:** 2020-08-12

**Authors:** L.-M. Sklarz, Y. S. Gladbach, M. Ernst, M. Hamed, C. Roolf, S. Sender, J. Beck, E. Schütz, S. Fischer, S. Struckmann, C. Junghanss, G. Fuellen, H. Murua Escobar

**Affiliations:** 1 Department of Medicine, Clinic III - Hematology/Oncology/Palliative Care, Rostock University Medical Center, Rostock, Germany; 2 Institute for Biostatistics and Informatics in Medicine and Ageing Research (IBIMA), Rostock University Medical Center, Rostock, Germany; 3 Faculty of Biosciences, Heidelberg University, Heidelberg, Germany; 4 Division of Applied Bioinformatics, German Cancer Research Center (DKFZ), National Center for Tumor Diseases (NCT) Heidelberg, Heidelberg, Germany; 5 Chronix Biomedical GmbH, Göttingen, Germany

**Keywords:** PIK3-inhibition, Acute lymphoblastic leukaemia, Idelalisib, Cytostatics, Drug combinations, Cytarabine, Dexamethasone

## Abstract

**Background:**

The introduction of combined conventional cytostatics and pathway-specific inhibitors has opened new treatment options for several cancer types including hematologic neoplasia such as leukaemias. As the detailed understanding of the combination-induced molecular effects is often lacking, the identification of combination-induced molecular mechanisms bears significant value for the further development of interventional approaches.

**Methods:**

Combined application of conventional cytostatic agents (cytarabine and dexamethasone) with the PI3K-inhibitor Idelalisib was analysed on cell-biologic parameters in two acute pro-B lymphoblastic leukaemia (B-ALL) cell lines. In particular, for comparative characterisation of the molecular signatures induced by the combined and mono application, whole transcriptome sequencing was performed. Emphasis was placed on pathways and genes exclusively regulated by drug combinations.

**Results:**

Idelalisib + cytostatics combinations changed pathway activation for, e.g., “Retinoblastoma in cancer”, “TGF-b signalling”, “Cell cycle” and “DNA-damage response” to a greater extent than the two cytostatics alone. Analyses of the top-20 regulated genes revealed that both combinations induce characteristic gene expression changes.

**Conclusion:**

A specific set of genes was exclusively deregulated by the drug combinations, matching the combination-specific anti-proliferative cell-biologic effects. The addition of Idelalisib suggests minor synergistic effects which are rather to be classified as additive.

## Background

Acute lymphoblastic leukaemia (ALL) is a malignant disease which is characterized by a clonal proliferation of lymphoid progenitor cells, most commonly of B-cells. ALL affects children as well as elderly individuals with a significantly different outcome. While children are reported to have a long-term survival probability of approximately 80% [1, 2], in adults relapse-free survival is lower than 30% [3]. Patients showing mixed-lineage leukaemia (*MLL*) rearrangements display even lower survival rates [4–7].

ALL therapy is currently dominated by the application of cytostatic agents, according to the current clinical practice guidelines [8]. However, severe side effects, development of drug resistance and relapse limit the therapeutic success [6].

The introduction of pathway specific tyrosine kinase inhibitors (TKI) such as imatinib [9] and immuno-therapeutics such as the anti-CD20 antibody Rituximab have advanced curative treatment in chronic leukaemia [10]. The phosphatidylinositol-4,5-bisphosphate 3-kinase (PI3K) represents a key molecule within the B cell receptor (BCR) pathway, and different TKIs are currently evaluated to target this kinase.

Idelalisib (IDEL) is a selective PI3K pathway inhibitor targeting the δ subunit [11, 12]. Mono and combined administration of IDEL were approved for the treatment of indolent non-Hodgkin lymphoma and chronic lymphocytic leukaemia [13–16]. However, post-marketing surveillance suggested increased mortality, caused by infections, as a side effect of IDEL. The molecular mechanisms leading to this observed side effect are not yet fully understood [17, 18].

IDEL preferentially targets the delta-subunit (PI3Kδ/p110δ) of the PI3K kinase, which plays a key role in signal transduction, cell proliferation and survival, energy metabolism, cellular motility, and cell cycle progression. The kinase is highly activated in several tumour types of different origins [13, 19, 20]. Consequently, PI3K is targeted in several novel therapeutic approaches [13, 21, 22] Table 1.

**Table 1:** Pathway analysis of RS4;11 and SEM exposed to AraC, DEX and IDEL and two drugs combined (AraC + IDEL, DEX + IDEL)

Pathway (regulation) AraC + IDEL vs. AraC vs. IDEL	Pathway: ranking position (corresponding genes)			Cell line
	AraC + IDEL	AraC	IDEL	
Retinoblastoma in Cancer (up)	10 (16)	90 (4)	87 (2)	RS4;11
TGF-beta signaling pathway (down)	19 (12)	31 (3)	90 (1)	
TGF-beta signaling PATHWAY (up)	17 (19)	37 (13)	–	SEM
SIDS susceptibility pathway (up)	19 (18)	35 (14)	–	
TNF-alpha signaling pathway (up)	20 (18)	34 (15)	–	

Pathway (regulation) DEX + IDEL vs. DEX vs. IDEL	DEX + IDEL	DEX	IDEL	Cell line
Proteasome degradation (down)	19 (28)	52 (10)	–	RS4;11
Cytoplasmic ribosomal proteins (up)	1 (42)	72 (5)	–	SEM
MicroRNAs in cardiomyocyte hypertrophy (up)	16 (18)	31 (10)	30 (1)	
Ectoderm differentiation (up)	19 (17)	39 (8)	–	
Retinoblastoma in cancer (down)	1 (55)	46 (10)	135 (1)	
Cell cycle (down)	2 (51)	32 (12)	56 (2)	
DNA replication (down)	5 (32)	80 (6)	–	
G1 to S cell cycle (down)	6 (32)	53 (9)	62 (2)	
DNA damage response (down)	13 (24)	37 (11)	58 (2)	

Ranking positions of the pathways and amount of corresponding genes (in parentheses) are represented

The introduction of TKIs such as IDEL enabled the evaluation of new drug combinations, potentially featuring lower doses of the individual drugs and thereby reducing side effects and drug resistance. In turn, understanding the respective combination modes of action is critical for a rational selection of the best candidate drugs [23]. Introduction of next-generation sequencing, such as whole genome and exome sequencing, and RNA-Sequencing provided profound knowledge of the disease acting molecular mechanisms. Especially RNA-Sequencing has been of considerable value as mRNA allowed to characterise drug combination action as well as drug combination induced effects [24].

Therefore, in the present study, the cell-biological and molecular effects of the PI3K-inhibitor IDEL in mono- and combined drug application with the conventionally used cytostatics cytarabine (AraC) and dexamethasone (DEX) on pro-B-ALL cells were investigated. Cell-biological assays analysing cell proliferation, metabolism, and apoptosis induction revealed combination-specific enhanced anti-proliferative effects of the combined drug applications. Comparative whole transcriptome sequencing analyses identified pathways and gene signatures specifically regulated by the respective drug combinations. Within the top-20 modulated pathways the “Retinoblastoma in cancer”, “TGF-b signalling”, “Cell cycle” and “DNA-damage response” were predominantly affected by the combination. In order to identify key player genes using these pathways, the top20 modulated were analysed revealing a gene set exclusively regulated by the drug combination in both cell lines. This gene set featured CYP3A4, STEAP1, SLITRK1, ACKR3, and CCL25. Some of these genes are reported to be deregulated in leukaemic cells. Thus, exclusively regulation by drug combination may explain the rather additive effects.

## Results

### IDEL enhances the anti-proliferative and anti-metabolic effect of AraC and DEX

**Fig. 1:**
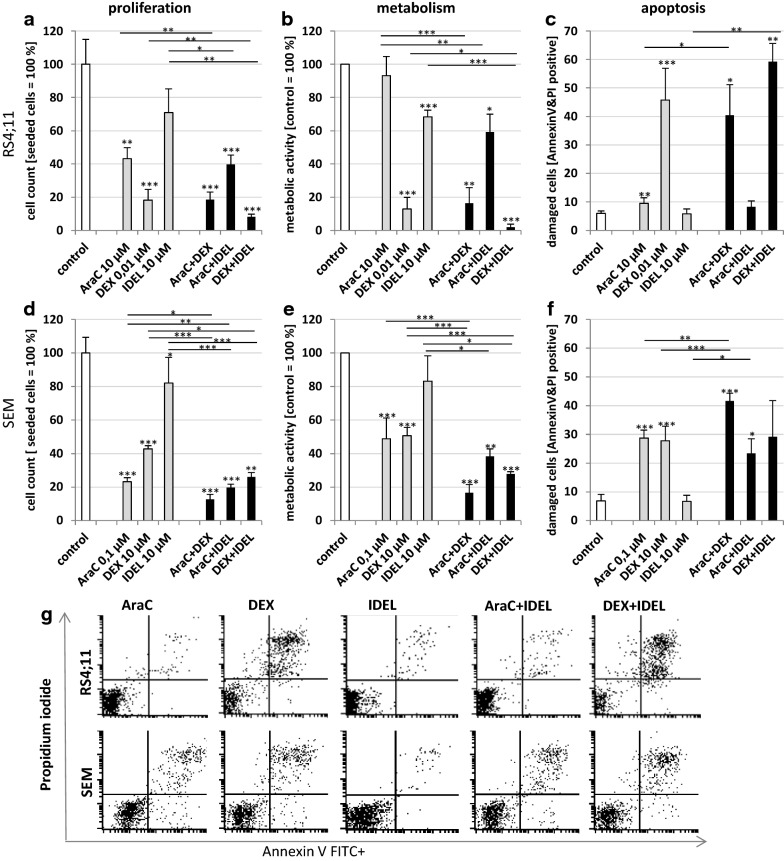
Pro-B ALL cell lines RS4;11 (**a**–**c**) and SEM (**d**–**f**) exposed with AraC, DEX and IDEL and two drugs combined (AraC+DEX, AraC+IDEL, DEX+IDEL) for 72 h. Influence of mono and combined application on (**a**, **d**) proliferation (cell count), (**b**, **e**) proliferation and metabolism (WST-1 proliferation assay) and (**c**, **f**) apoptosis (Annexin V/PI- staining). A pairwise students t-Test compared to control cells and single compounds reveals significance: *p ≤ 0.05, **p ≤ 0.01, ***p ≤ 0.001, [n ≥ 3]. **g** Plots of apoptotic (Annexin V FITC+ and Propidium iodide-) and necrotic cells (Annexin V FITC + and Propidium iodide +) detected by flow cytometry analysis at 72 h. Data are representative of three independent experiments. Further plots are represented in the Additional file 1: FACS plots

In RS4;11 cells, enhanced effects on proliferation inhibition were observed for combinations of AraC + IDEL (40 ± 6%) and DEX + IDEL (8 ± 2%), compared to the respective mono drug applications (AraC: 43 ± 7%, DEX: 18 ± 6%, IDEL: 71 ± 14%, control: 100%) (Fig. 1a). Comparison of cell count and metabolic activity (WST-1-Assay, Fig. 1b) revealed a reduction in cell numbers while barely decreasing metabolic activity for AraC and AraC + IDEL (93 ± 12% and 59 ± 11%).

In SEM cells, all combinations (AraC + DEX: 13 ± 3%, AraC + IDEL: 20 ± 2%, DEX + IDEL: 26 ± 2%) resulted in an enhanced anti-proliferative effect compared to the respective mono applications (AraC: 23 ± 2%, DEX: 43 ± 2% or IDEL: 82 ± 15%) (Fig. 1d). Akin to RS4;11, the incubation with AraC (49 ± 12%) and AraC + IDEL (38 ± 4%) resulted in a decreased metabolic activity (Fig. 1e). The observed reduction of metabolism did not match the observed reduction in cell numbers.

### IDEL boosts the apoptotic effect of DEX in RS4;11

Incubation with DEX + IDEL resulted in a significantly higher apoptosis rate (59 ± 6%), compared to the respective mono substance application (DEX: 46 ± 11%, IDEL: 6 ± 2%, control: 6 ± 1%) in RS4;11 cells (Fig. 1c). In SEM, only the combination AraC + DEX resulted in an increased amount of early and late apoptotic cells (42 ± 3%), compared to the respective mono substances (AraC: 29 ± 3%, DEX: 28 ± 5%, control: 7 ± 2%) (Fig. 1f). In Fig. 1g are exemplarily the plots of the flow cytometry analysis. Additionally, all plots are shown in the supplementary file (Additional file 1: FACS plots).

In summary, biological assays revealed enhanced anti-proliferative effects triggered by combined application of IDEL with AraC and DEX, respectively. Therefore, we further investigated drug exposure induced effects on gene and pathway regulation by RNA-Sequencing for all drug combinations and mono applications.

### Combined drug application of IDEL with AraC and DEX induces enhanced changes in gene expression

#### Drug combination induces an enhanced amount of regulated genes

**Fig. 2:**
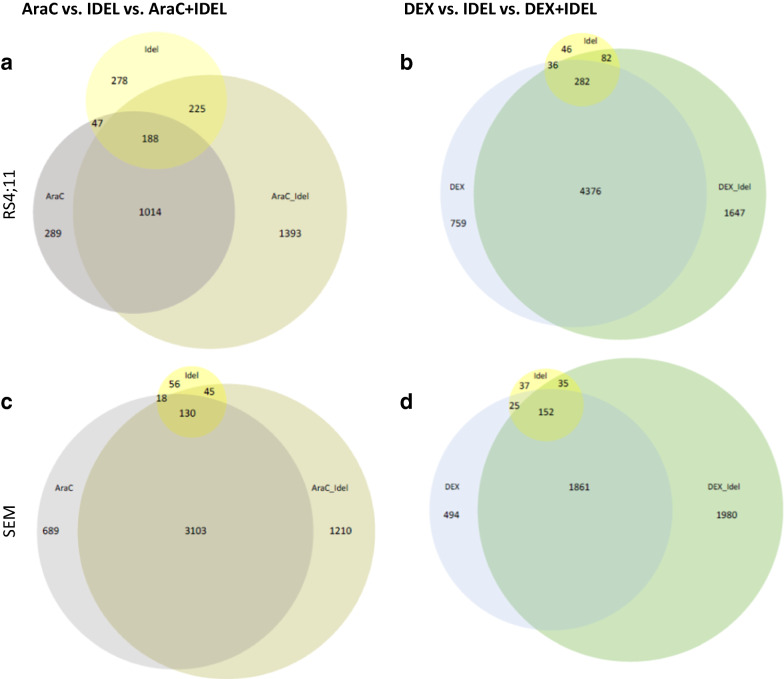
Pro-B ALL cell lines RS4;11 (**a**, **b**) and SEM (**c**, **d**) exposed with AraC vs. IDEL vs. AraC+IDEL and DEX vs. IDEL vs. DEX+IDEL. Venn-diagrams represent the differential expressed genes (DEG) of each sample and there overlap among these samples

In RS4;11, 2820 genes were differentially expressed by AraC + IDEL exposure compared to the respective control cells, while mono application of AraC modulated 1538 and IDEL 738 genes (see Additional file 2: supplement tables). Thereby, an overlap of 188 genes in all three conditions was identified (Fig. 2a).

Also, DEX + IDEL exposure modulated more genes (6387) compared to DEX (5453) or IDEL (446). Here, an overlap of 282 genes was detected by both mono and as well as combined drug application (Fig. 2b).

In SEM, the AraC + IDEL combination led akin to RS4;11 to a stronger gene modulation (4488) compared to AraC (3940) and IDEL (249). Thereby, 130 genes were modulated by both, the mono and the combined drug application (Fig. 2c). Moreover, DEX + IDEL exposure resulted in a higher amount of regulated genes (4029) in comparison to their respective mono applications (DEX: 2532; IDEL: 249). All three conditions showed an overlap of 152 genes in total.

In summary, for both cell lines and both drug combinations, the total number of genes modulated by these combinations exceeds the absolute number of the respective mono application. Especially, the conditions AraC vs. AraC + IDEL and DEX vs. DEX + IDEL showed a high overlap of modulated genes. Heatmaps of the top-100 regulated genes are shown in the Additional file 3: supplement figures.

However, the comparison of the up- and downregulated genes revealed a higher amount of upregulated genes by AraC + IDEL exposure in both cell lines. Moreover, DEX + IDEL led nearly to a similar amount of up- and downregulated genes in both cell lines.

#### Drug combinations induced stronger changes of gene expression levels

In RS4;11, we observed a higher range of gene expression level changes by AraC + IDEL (− 3.56 to 4,98) in comparison to AraC (− 2.11 to 5.03) and IDEL (− 3.51 to 3.92). An enhanced range was also observed by exposure with DEX + IDEL (− 7.49 to 11.98) in contrast to the respective mono application (DEX: − 5.1 to 11.65; IDEL: − 3.59 to 3.5).

As with RS4;11, a higher range was observed in SEM cells by combined drug incubation with AraC + IDEL (− 4.99 to 8.35) compared with mono application (AraC: − 4.09 to 8.45, IDEL: − 1.84 to 1.9). Similar effects were detected for DEX + IDEL (− 6.88 to 9.66) exposure (DEX: − 5.65 to 9.67, IDEL: − 1.84 to 1.9).

#### AraC + IDEL and DEX + IDEL modulated combination specific gene sets

In RS4;11, AraC + IDEL exposure led to a set of 982 genes, which were exclusively regulated by this combination but not by DEX + IDEL. In contrast, DEX + IDEL led to selective modulation of 4549 genes which were only regulated by this combined drug application (see Additional file 2: supplement tables).

In SEM, AraC + IDEL led to an exclusively regulation of 2589 genes which were not modulated by the other drug combination. Exposure to DEX + IDEL resulted led to a set of 2130 exclusively effected genes, which were not regulated by AraC + IDEL (see Additional file 2: supplement tables).

In summary, the combined application of IDEL with AraC or DEX resulted in regulation of an exclusively gene set and also in higher gene expression levels. Further, the specific combinations induce characteristic expression changes.

### Combined drug application of IDEL with AraC or DEX leads to combination specific pathway modulation

A combination of AraC + IDEL or DEX + IDEL led to specifically enhanced pathway modulation compared to the respective mono applications. Absolute numbers of genes included in the respective pathways were increased. In the following, we focussed on pathways that did not rank within the top-30 deregulated pathways within the mono applications but ranked in the top-20 pathways during combined setup (overview in Table 1, a complete listing table in the Additional file 4: xls-file, section “Additive pathways + genes”).

#### AraC + IDEL modulates TGF-beta signalling in RS4;11 and SEM and further three cell line specific pathways

In RS4;11 cells, the combination of AraC + IDEL led to 16 upregulated genes corresponding to the “Retinoblastoma in Cancer” pathway while the respective mono applications modulated only four (AraC) respectively two (IDEL) genes respectively. Accordingly, the pathway ranked on position 10 of the top modulated pathways (AraC: position 90; IDEL: position 87). Further, “TGF-beta signalling” showed more downregulated genes (12) after AraC + IDEL exposure compared to the mono applications (AraC: 3 genes, IDEL: 1 genes). Combined pathway ranking on position 19 (AraC: position 31, IDEL: position 90).

In SEM, with respect to the modulation of the “TGF-beta signalling” pathway a higher number of downregulated genes (19) was found by AraC + IDEL in comparison to the respective mono applications (AraC: 13, IDEL: 0). This pathway ranked on position 17 for the combined application (AraC: position 37, IDEL: N.A.).

Further, “TNF-alpha signalling” was found on position 20 of the top upregulated pathways (AraC: position 34, IDEL: N.A.) modulating 18 combination specific genes compared to AraC (15 genes) and IDEL (0 genes). Accordingly, TGF-alpha signalling was found to be a major target of IDEL combination induced pathway modulation.

Further, upregulation of 18 genes corresponding to the “SIDS susceptibility” pathway was observed by AraC + IDEL exposure compared to the respective mono applications (AraC: 14 genes, IDEL: 0 genes). This pathway was found at position 19 of the list of the top-20 regulated pathways (AraC: 35, IDEL: N.A.).

#### DEX + IDEL exposure induces extensive “Cytoplasmic ribosomal proteins”, “Retinoblastoma in Cancer” and “Cell cycle” pathway deregulation

While AraC + IDEL modulated four different pathways in total in both cell lines, the induced observed modulation by the addition of IDEL to DEX resulted in a high number of combination specific pathway deregulations. In total, nine different pathways were modulated by DEX + IDEL in both cell lines. Interestingly, eight of nine pathways were found to be significantly deregulated in SEM (3 upregulated, 5 downregulated).

Thereby, the total number of modulated genes exceeded the number of genes modulated by the respective mono application in pathways such as “Cytoplasmic ribosomal proteins” (upregulated), “Retinoblastoma in Cancer” and “Cell cycle” (both downregulated). While 42 genes belonging to the “Cytoplasmic ribosomal proteins” pathway were found upregulated by DEX + IDEL, only five genes were found upregulated by DEX and 0 by IDEL. A similar pattern was observed for the “Retinoblastoma in Cancer” pathway (DEX + IDEL: downregulation of 55 genes vs. DEX: 10 genes vs. IDEL 1 gene) as well as for the “Cell cycle” pathway [25] (DEX + IDEL: 51 genes vs. DEX: 12 genes vs. IDEL: 2 genes).

Further pathways, showing a similar combination specific enhanced modulation were pathways such as “MicroRNAs in cardiomyocyte hypertrophy”, “Ectoderm differentiation”, “DNA replication”, “G1 to S cell cycle” and “DNA damage response” in SEM cells and the “Proteasome Degradation” pathways in RS4;11 (detailed listing in Additional file 4: xls-file, section “Additive pathways + genes”).

In summary, the additional application of IDEL enhances the observed effects of AraC and DEX.

#### IDEL combination modulated pathways showed characteristic gene deregulations

DEX + IDEL as well as AraC + IDEL combination induced specific gene modulation not found in either of both mono applications. In total AraC + IDEL led to an exclusive modulation of 33 genes annotated with the four drug combination specific pathways (log fold-change: − 0.79 to 1.39). The DEX + IDEL combination induced exclusive modulation of in total of 124 genes within the 9 drug combination specific modulated pathways (log fold-change: − 1.11 to 1.12). Exemplarily, DEX + IDEL exposure led to exclusive modulation of the Cell Division Cycle 25 A (*CDC25A;* log fold-change: − 1.11*)* [25], Cell Division Cycle 6 (*CDC6;* log fold-change: − 1.05) [26] and Myosin light-chain kinase gene (*MYLK;* log fold-change: 1.12) [27] genes.

A detailed listing of all affected genes of both cell lines can be found in the Additional file 4: xls-file, section “Additive pathways + genes”.

#### IDEL combination specific pathways show enhanced gene expression regulation

As described above, the addition of IDEL to AraC or DEX led to exclusive gene regulations as well as increased gene numbers belonging to to the top deregulated pathways. Additionally, the respective combinations led to enhanced log fold-changes for a set of specific genes (summary is given in the Additional file 2: supplement tables). Thereby, the respective range in the combinations exceeded the respective mono application (detailed listing in the Additional file 4: xls-file, section “Additive pathways + genes”. As mentioned before, AraC + IDEL led to a drug combination specific pathway modulation of four pathways. Further investigation revealed a deregulation of 84 genes by AraC + IDEL (log fold-change range: − 0.81 to 4.04), while AraC deregulates 54 genes (log fold-change range: − 0.85 to 5.25) and IDEL 4 genes (log fold-change range: − 0.58 to 0.71). Incubation with DEX + IDEL led to a deregulation of nine drug combination specific modulated pathways. From these pathways, DEX + IDEL deregulated 202 genes (log fold-change range: − 5.12 to 7.92), while DEX deregulated 80 genes (log fold-change range: − 3.46 to 7.62) and IDEL none. Exemplarily, genes such as Aristaless Related Homeobox (*ARX)* [28] and Zinc Finger And BTB Domain Containing 16 (*ZBTB16)* [29] were upregulated by DEX application (ARX: log fold-change 4.97; ZBTB16: log fold-change 7.62) and stronger deregulated by the drug combination DEX + IDEL (ARX: log fold-change 5.21, ZBTB16: log fold-change 7.92). For a detailed comparison of the combined and mono application induced expression changes see Additional file 4: xls-file, section “Additive pathways + genes”. AraC exposure led to an upregulation of Distal-Less Homeobox 2 (*DLX2)* [30] (log fold-change 2.59), while the addition of IDEL induced a log fold-change 3.63 for the combined application.

### Top-20 drug combination modulated genes revealed combination specific modes of action

To further explore combination specific acting mechanisms, the top-20 deregulated genes (pathway independent log fold-changes, combined drug exposure) were compared to the corresponding expression values of the respective mono applications (Additional file 4: xls-file, section “RS4;11-top20 genes A+ID+I” and “SEM-top20 genes A+ID+I”). Akin to the observed exclusive gene modulations within the top ranking pathways, comparable effects were found when analysing the general pathway independent top-20 deregulated genes.

#### AraC + IDEL modulates histone genes predominantly

AraC + IDEL exposure led in both cell lines to a downregulation of different histones. Thereby, *HIST1H2BO* was found the only histone which ranked within the top-20 downregulated genes for both cell lines.

In RS4;11, twelve histones belong to the general top-20 downregulated genes. Thereby, the observed log fold-change of the histones *HIST1H1E*, *HIST1H2AH, HIST1H1D, HIST1H2BM,* and *HIST1H3B* were found stronger deregulated given the observed log fold-changes compared to both respective mono applications (range log fold-change: AraC + IDEL − 3.14 to − 2.69 vs. AraC − 1.96 to − 1.75 vs. IDEL − 2.36 to − 1.87). Further, the histones *HIST1H3I* and *HIST1H3F* were exclusively found to be downregulated by AraC and AraC + IDEL, while not being affected by IDEL. Interestingly, both histones were more affected by AraC + IDEL (log fold-change: − 3.26 and − 2.90) compared to AraC (log fold-change: − 1.99 and − 1.58). Further, the histones *HIST1H2BF* (log fold-change: − 3.37), *HIST1H2AJ* (log fold-change: − 3.35), *HIST12AD* (log fold-change: − 3.23), *HIST1H3G* (log fold-change: − 2.52) and *HIST1H2BO* (log fold-change: − 2.81) were found to be exclusively downregulated by the AraC + IDEL.

In SEM, histones as *HIST1H2BB* (log fold-change: − 3.35) and also *HIST1H2BO* (log fold-change: − 3.00) were only affected by AraC + IDEL. While histones as *HIST1H4B, HIST1H2BE, HIST1H4J, HIST1H2BG,* and *HIST1H3A* were downregulated by AraC and stronger affected by AraC + IDEL (log fold-changes are detailed listed in the Additional file 4: xls-file, section “SEM-top20 genes A+ID+I”).

In addition to the mentioned histones, both cell lines showed a specific pattern of the remaining combination specific top-20 deregulated genes. In RS4;11, downregulation of the genes Small Nucleolar RNA 12 (*SNORA12* [31]; log fold-change − 2,84), Nucleoside Diphosphate Kinase 1/2 (*NME1*-*NME2* [32]; log fold-change − 2.77), and Eukaryotic Translation Initiation Factor 5A-Like 1 (*EIF5AL1*; log fold-change − 2.73) and an upregulation of CAMP Responsive Element Binding Protein 3 Like 3 (*CREB3L3* [33]; log fold-change 4.24) and Transmembrane And Immunoglobulin Domain Containing 2 (*TMIGD2* [34]; log fold-change 3.95) were found.

In SEM, Cytochrome P450 Family 3 Subfamily A Member 4 (*CYP3A4* [35]; log fold-change − 4.11), Six Transmembrane Epithelial Antigen Of The Prostate 1 (*STEAP1* [36]; log fold-change − 3.03) and Potassium Voltage-Gated Channel Subfamily J Member 1 (*KCNJ1* [37]; log fold-change − 4.99) represents genes which were found only downregulated by the combination AraC + IDEL.

#### DEX + IDEL leads to regulation of similar top-20 modulated genes in both cell lines

In contrast to AraC + IDEL, the exposure with DEX + IDEL induced a higher number of genes commonly deregulated in both cell lines. In both cell lines, two genes were found upregulated as well as eight downregulated ranking within the top-20 deregulated genes.

Ring Finger Protein 175 (*RNF175)* was downregulated in both cell lines by DEX and DEX + IDEL exposure. In RS4;11, *RNF175* was significantly more affected by the drug combination (DEX: log fold-change − 2.62, DEX + IDEL: log fold-change − 7.49). In SEM, *RNF175* showed a similar expression level with DEX + IDEL exposure (log fold-change − 4.58) as with DEX (log fold-change − 4.72). Also *ZBTB16* (described above), Olfactory Receptor Family 7 Subfamily A Member 5 *ORC7A5* and Olfactory Receptor Family 7 Subfamily C Member 1 *OR7C1* were upregulated and more affected by the drug combination in both cell lines.

Additionally, some genes were exclusively deregulated by drug combination. In RS4;11, Leucine-Rich Repeat-Containing Protein 12 (*SLITRK1* [38]; log fold-change − 4.85) and Matrilin 4 (*MATN4* [39]; log fold-change − 4.77) were only downregulated by the drug combination DEX + IDEL. In SEM, Atypical Chemokine Receptor 3 (*ACKR3* [40]; log fold-change − 4.41) and C–C Motif Chemokine Ligand 25 (*CCL25* [41]; log fold-change − 5.54) were also only downregulated by DEX + IDEL. In both cell lines, the top-20 upregulated genes did not contain any gene which was exclusively regulated by the drug combination DEX + IDEL. The here reported genes comprise a short overview and more genes are listed in the Additional file 3: xls-file, section “RS4;11-top20 genes A+ID+I”, “SEM-top20 genes A+ID+I”.

### Discussion

The combined application of IDEL and AraC or DEX resulted in enhanced anti-proliferative effects depending on the addressed cell line. The combination led to enhanced anti-proliferative effects on the cellular level, characteristic gene regulation, and expression. Thereby, the specific exclusively regulated genes and pathways were identified in both *MLL*-positive pro-B-ALL cell lines. The focus here is on mono- and combined therapy of maximum two agents in cell lines and adds insights into the previously gained knowledge of expression profiling as well as fusion gene detection in patients with B-ALL using standard treatment regimen containing AraC and DEX [42, 43].

#### Addition of IDEL to AraC results in pronounced anti-proliferative effects independent from AraC-sensitivity

A different AraC-sensitivity characterises both cell lines. While 10 µM AraC exposure reduces RS4;11 proliferation to approx. half, SEM proliferation is inhibited to nearly 25% by the 100-fold lower concentration. The addition of IDEL induced in both cell lines, independently from their characteristic AraC-sensitivity, an enhanced decrease of proliferation. Interestingly, AraC exposure led to an increase of metabolic activity in both cell lines, while the addition of IDEL leads to a proportional ratio of remaining cells and corresponding metabolic activity. Further, the addition of IDEL initiates an increase of the number of regulated genes and stronger modulated gene expression levels. Further, the addition of IDEL led to a modulation of genes belonging to the “TGF-beta signalling” pathway in both cell lines. This pathway is an essential regulator of proliferation, differentiation, migration, and cell survival [44]. Additionally, several genes regulating cellular key processes as proliferation and cell cycle were found regulated explicitly by the addition of IDEL. Exemplarily, various histones with direct effect on DNA-packaging were found downregulated and thus influence DNA-replication. Further, the elongation factor *EIF5AL1* [45] was found exclusively downregulated by AraC + IDEL in RS4;11. Both mechanisms show a specific enhancement of cell division critical check-points, which could be mediators of the observed cellular response.

Genes as *SNORA12, NME1*-*NME2, CYP3A4,* and *STEAP1* were exclusively downregulated by AraC + IDEL. These genes are described to be found overexpressed in cancer of different origins. While *SNORA12* is found upregulated in lung cancer [31], *NME1*-*NME2* upregulation is described to promote the survival of AML cells [32]. *STEAP1* overexpression is detected in different cancer types [46] and was associated with a poorer prognosis for AML, multiple myeloma, diffuse large B cell lymphoma, and colorectal cancer [36]. Accordingly, the observed exclusively downregulation potentially represents a molecular mechanism resulting in the enhanced anti-proliferative effects of AraC + IDEL. Further, we detected a selective downregulation of *CYP3A4.* CYP3A4-A-290G polymorphism that resulted in overexpression, was found in many acute myeloid leukaemia (AML) samples [47]. Additionally, an overexpression in breast cancer [35] had been detected. *CYP3A4* is responsible for the detoxification of more than 50% of the drugs [47]. On the other hand, we discovered an exclusive downregulation of *KCNJ1.* This gene is reported to inhibit proliferation and metastasis in renal cell carcinoma [37]. Currently, data of *KCNJ1* for leukaemia is missing. The examined downregulation of these genes by AraC + IDEL may contribute to the more potent effect of the drug combination in comparison to the respective mono application.

While the previous represents the loss of pro-oncologic cellular features, also gain of function modulations were observed, leading to the enhanced anti-proliferative molecular mechanism. Exemplarily, *CREB3L3* was found exclusively upregulated while *DLX2* expression was found stronger upregulated by the combination. *CREB3L3* overexpression suppresses proliferation in hepatoma cells [48] and has been described to be downregulated in hepatocellular carcinoma [33]. *DLX2* is reported to be downregulated in paediatric precursor B-ALLs carrying *MLL*-rearrangement and may be induced during metabolic stress-induced necrosis [30].

These functional gene modulations represent candidate mechanisms mediating the observed enhanced anti-proliferative effects in the ALL cell lines.

Interestingly, in ALL similar cell biological effects were observed, showing that the addition of PI3K- or mTOR inhibitors to AraC was able to induce enhanced anti-proliferative effects in vitro [49]. Lately, comparative observations were described for the combination of the PI3K-δ inhibitor Puquitinib with AraC in an AML xenograft model [50].

#### Addition of IDEL to DEX leads to enhanced anti-proliferative effects in glucocorticoid-sensitive and -resistant cells

The addition of IDEL to DEX resulted in an enhanced anti-proliferative effect and higher apoptosis rates in glucocorticoid (GC)-resistant and –sensitive pro-B-ALL cell lines. Both cell lines are characterized by a translocation between chromosome HSA4 and HSA11. In general, the presence of this translocation is associated with GC-resistance and often observed in cases of relapses. However, only SEM cells (established from a 5-years-old girl at relapse) show reduced sensitivity to DEX, while RS4;11 (established from a 32-year-old woman at relapse) are considered as highly sensitive. While in SEM, 10 µM DEX exposure inhibited the rate of proliferation to approx. a half, RS4;11 proliferation was found decreased to nearly 20% by the 1000-fold diluted concentration. The addition of IDEL to DEX led to a strongly DEX-sensitizing effect on both cell lines. SEM proliferation was further reduced to approx. 25%, while RS4;11 proliferation was reduced to nearly 10%. Additionally, the observed apoptosis inducing effect of DEX was found increased by the addition of IDEL in both cell lines.

Akin to the effects observed for AraC + IDEL, the addition of IDEL to DEX induced specific pathways modulations and also induced exclusive gene expression influencing key regulators such as cell cycle and DNA-replication. Thereby, key mediators as *CDC25A* and *CDC6* were found exclusively downregulated by the DEX + IDEL combination. *CDC25A* overexpression had been found in breast cancer [25] and *CDC6* overexpression in cervix, lung and brain cancer [26]. Thus, potentially the observed exclusively downregulation represents a molecular mechanism leading to the enhanced anti-proliferative effects of DEX + IDEL.

Additionally, genes such as *ZBTB16* and *MYLK* were found exclusively upregulated by the drug combination. The tumour suppressor *ZBTB16* was found to be downregulated in non-small-cell lung cancer, decreased expression levels may contribute to cell survival, and overexpression was found to increase apoptosis and inhibit proliferation [51]. Additionally, malignant mesothelioma cell data indicated that the downregulation of *ZBTB16* might promote cell survival [29]. Also, low levels of *MYLK* were found in non-small cell lung cancer. Interestingly, higher expression levels of *MYLK* were found in samples of patients with chronic lymphocytic B-cell leukaemia in the lymph nodes in comparison to the respective bone marrow, peripheral blood, and healthy donors [52]. The different tissue types may explain this. Currently, data for ALL are missing. The examined upregulation of *ZBTB16* and *MYLK* by DEX + IDEL may contribute to the more potent effect of the drug combination in comparison to the respective mono application.

Further, *OR7A5* and *OR7C1* were upregulated in our experiments and more regulated stronger by the drug combination in both cell lines. Olfactory Receptors (OR) are reported to have different expression levels in tissue of various origin, and OR7C1 represents a prognostic biomarker in colon cancer [53]. Manteniotis et al. [54] presents data on myelogenous leukaemia cells, which suggests that OR2AT4 stimulation resulted in reduced proliferation and enhanced apoptosis. Currently, data of *OR7A5* and *OR7C1* in leukaemia are not reported.

Furthermore, we detected genes like *SLITRK1, ACKR3,* and *CCL25,* which were exclusively downregulated by DEX + IDEL. All these genes are reported to be upregulated in leukaemia, and the detected downregulation may explain the examined enhanced anti-proliferative effect by the drug combination. It is reported that ALL cells express more *SLITRK1,* and it may play a role in hematopoiesis and possibly leukemogenesis [55]. *ACKR3* is upregulated in ALL [56], as well as AML, and is necessary for colonization of the draining lymph node in diffuse large B cell lymphoma [40]. *CCL25* promotes with Wingless-Type MMTV Integration Site Family Member 5A *WNT5A,* the cell migration, invasion, and metastasis of adult T-ALL [41].

On the other hand, also MATN4 was exclusively downregulated by DEX + IDEL.

*MATN4* is highly expressed in long-term hematopoietic stem cells and is strongly downregulated in response to proliferative stress [39]. Data for progenitor cells are lacking.

Recently, Kruth et al. [15] characterized the mono drug application in B-ALL cells and the combined drug application of DEX with IDEL in pre-B-ALL cells in vitro and patient derived ALL-xenografts in vivo. Also, they tested mono DEX in RS4;11 (pro-B-ALL) and detected repression of *ITGA4, IL7R,* and *BCL6*. In both pro- and pre-B-ALL cells, DEX exposure leads to the downregulation of *BCL6*. However, in our pro-B-ALL RS4;11 cells, the combined drug incubation DEX + IDEL suppresses this effect without neutralizing it entirely, while in SEM, the drug combination enhanced the downregulation of *BCL6. BCL6* is expressed in pre-B cells, acts as a transcription factor, and is necessary for maturation of pro- to pre-B cells [15]. However, occasionally, pro-B cells were reported to express *BCL6* [57]. In lymphoma, high expression levels of *BCL6* were reported to be associated with pro-survival and proliferation functions [58]. Thus, downregulation aspires to be a direct interventional effect of the applied components of minor influence by the combination. In SEM cells, DEX exposure also leads to a downregulation of *IL7R,* and the combination DEX + IDEL suppresses this effect without neutralizing it. Interestingly, IL7R fulfils a crucial role in the proliferation of pro- and large pre-B cells. Accordingly, reduced expression of IL7R was correlated to less proliferation and transcription of pro-survival genes in pro-B cells [59]. Pro- and pre-B-ALL differ in their grade of maturation and their presentation of surface molecules and receptors. These differences can have a major influence on the cellular response, e.g., pro-B cells are typically characterized by carrying an IL7R, while only large pre-B cells are characterized by this receptor [59].

Interestingly, the observed increased anti-proliferative effects in pre- and pro-B-ALL cells showed further similarities. Comparison of the data of pro- and pre-B-ALL showed, in our RS4;11 cells activation of the pro-apoptotic active *BCL2L11* by DEX exposure. However, in our used pro-B-ALL cell lines SEM and RS4;11, higher gene expression modulations were found induced by DEX + IDEL (see Additional file 4: xls-file, section "comparison Kruth et al.”). Despite the observed common induced effects in pro- and pre-B-ALL cells, the herein analysed cell lines showed characteristic differences. In pre-B-ALL, DEX leads to the repression of genes related to the BCR-signalling (CD79B, CSK, FYN, BTK, PIK3CD, PIK3C2B, and PIK3R2). Also, we could observe some tendencies, but could not detect this strong regulation of these genes. Exemplarily, in pro-B-ALL, an upregulation of *CD79B* in DEX exposed RS4;11 cells was observed, and the combined drug incubation DEX + IDEL suppresses this effect without neutralizing it completely. This difference, in particular, is interesting since CD79B composes part of the BCR and can phosphorylate SYK (Spleen Tyrosine Kinase) and LYN (Proto-Oncogene, Src Family Tyrosine Kinase) kinases. SYK is currently addressed in different clinical trials e.g. by the inhibitor Entospletinib for CLL, AML, ALL, and Lymphoma [60].

The herein employed RNA-Sequencing allows the superior analysis of transcriptomic regulation and, thus, additional significant genes modulations of, e.g., *SLITRK1, MATN4, ACKR3,* and *CCL25*. Also more regulated pathways were revealed.

Furthermore, our analysis showed that the DEX + IDEL combination deregulated pathways exclusively and significantly, such as “Proteasome Degradation”, “Cell cycle”, “DNA-Replication”, and “DNA-damage response”.

Further, Silveira et al. [21] combined an alternative pan-PI3K inhibitor (AS605240) with the glucocorticoid Prednisolone and detected enhanced anti-leukemic effects in T-ALL cells compared to the respective mono agents in vitro. Akin to this, Spijkers-Hagelstein et al. [7] examined in MLL-rearranged cell lines (among others SEM) beneficial effect in glucocorticoid-resistant cells as well by inhibition of PI3Kα,δ and β by the usage of LY294002.

In RS4;11, a difference in gene regulation of the IDEL samples was detected between both drug settings. A batch effect might explain this, but similar effects and tendencies were detected in general.

## Conclusion

In general, our data showed in mono application settings stronger anti-proliferative effects of AraC and DEX in comparison to the PI3K-inhibitor on the cells and on the molecular biological processes (gene expression, pathway regulation, proliferation, metabolism, and apoptosis) in the examined pro-B-ALL cells in vitro–comparable analyses are to the best of our knowledge not documented in literature. However, the minor induced modulations of IDEL led to a significant additive effect in the combined application scheme with the conventional cytostatic agents. Thus, drug combination resulted in more anti-proliferative effects, modulation of specific pathways, stronger gene expression level modulation and regulation of exclusive genes. The identification of the acting molecular mechanisms allows insights into cell biologic drug response and thus provides the basis for an intelligent regimen design to optimise therapeutic success.

## Materials and methods

### Cell lines and culture conditions

The pro-B ALL cell lines RS4;11 and SEM were purchased from “Deutsche Sammlung von Mikroorganismen und Zellkulturen GmbH“(Braunschweig, Germany) and cultured according to the manufacturer´s protocol. RS4;11 was grown in Alpha-MEM Medium (Biochrom GmbH, Berlin, Germany) and SEM in Iscove’s MEM Medium (Biochrom GmbH, Berlin, Germany) both supplemented with 10% heat-inactivated fetal bovine serum (Biochrom GmbH, Berlin, Germany) and 1% penicillin/streptomycin (Biochrom GmbH, Berlin, Germany). Cells were cultivated at 37 °C in a humidified atmosphere with 5% CO_2_ mostly placed in T175–tissue culture flasks (Greiner Bio-One GmbH, Frickenhausen, Germany) in downright position.

### Cytostatics and inhibitors

IDEL was purchased from Selleck Chemicals (Absource Diagnostics GmbH, München, Germany) and dissolved in dimethyl sulfoxide ((DMSO), Sigma–Aldrich Chemie GmbH, Steinheim, Germany) to a 10 mM stock solution and stored at − 80 °C.

DEX (8 mg inject Jenapharm^®^) was purchased from mibe GmbH (Brehna, Germany) and AraC (100 mg/ml) from cell pharm GmbH (Bad Vilbel, Germany).

### Drug application experiments

Cells were treated with drug concentrations similar to clinical settings and allowing a threshold of above 30% living cells after 72 h of incubation or alternatively a maximum of 10 µM. After an incubation time of 72 h we analysed effects of single (AraC, DEX, IDEL) and combined (AraC + DEX, AraC + IDEL, DEX + IDEL) application on proliferation (trypan blue staining) as well as metabolism (WST-1 assay), apoptosis/necrosis (AnnexinV/PI staining) as well as gene expression levels. 0.333 × 10^6^ cells per millilitre were used for all experiments. The cytostatics were diluted in phosphate–buffered saline (PBS) for the inhibitory experiments. Control cells were incubated with their respective medium containing the same concentration of DMSO (vs. IDEL) or PBS (vs. AraC and DEX) as the cells treated with the different drugs.

### Proliferation and metabolism studies

*Cell count:* RS4;11 and SEM cells were seeded at a density of 0.5 × 10^6^ cells per 1.5 ml in 24–well plates (Greiner Bio–One GmbH, Frickenhausen, Germany) for a cell count analysis. After 72 h incubation, the cells were harvested and washed in PBS (10 min, 180 g, 4 °C) and cell counts were determined using trypan blue staining (Sigma-Aldrich Chemie GmbH, Steinheim, Germany).

*WST*–*1 proliferation assay:* RS4;11 and SEM cells were seeded in biological triplicates at a density of 5 × 10^4^ cells per 150 µl per well in 96–well plates. Metabolic activity was analysed via tetrazolium compound WST–1 (TaKaRa Bio Inc., Kusatsu, Japan). After 72 h, the cells were incubated with 15 µl WST–1 for up to 3 h. In brief, the mitochondrial dehydrogenases reduce the light right and soluble WST–1 the dark red formazan. The amount of formazan dye directly correlates to the number of active metabolic cells, and a colour change can be measured by photometer with an absorbance at 450 nm and a reference wavelength at 750 nm. The absorbance of pure culture medium with added WST–1 was used as background control.

The comparison of the data of the cell count and the WST-1 assay give a hint about the metabolic activity of the cells.

### Early and late apoptosis analysis

Early and late apoptosis were determined by staining with Annexin V FITC (BD Biosciences, Heidelberg, Germany) and Propidium iodide ((PI) Sigma Aldrich, St. Louis, USA) according to the manufacturer´s protocol and analysed by flow cytometry.

Briefly, cells were harvested from 24-well plates and washed (10 min, 180 g, 4 °C) twice with PBS. After resuspending the cell pellet in 100 µl Annexin–binding buffer (BD Biosciences, Heidelberg, Germany), 5 µl Annexin V FITC was added and incubated for 15 min in the dark at room temperature. 400 µl binding buffer were added to the cell suspensions and stained with PI (20 µg/ml) immediately before measurement. Unstained and single strained control cells were included in each experiment. Measurements were performed using FACSCalibur (BD Biosciences, Heidelberg, Germany) and CellQuest Pro software (BD Biosciences, Heidelberg, Germany, Version 4.0.2).

### RNA–isolation and transcriptome analysis

Cells were cultivated with either single drugs or a combination for 72 h in T175–tissue culture flasks. Cells were harvested and washed (10 min, 180 g, 4 °C) in PBS three times. Total RNA was extracted using the miRNeasy Mini Kit (Qiagen, Hilden, Germany) as described in the Quick–Start Protocol. 500 µl Buffer RWT and 350 µl Buffer RPE were added twice after performing the DNase digest. The RNA quality and quantity were assessed using the NanoDrop Spectrophotometer ND–1000 (Peqlab Biotechnologie GmbH, Erlangen, Germany, Version 3.7.1).

Each treatment condition was prepared and sequenced in triplicates. For the preparation of sequencing libraries, 1 µg total RNA with RNA integrity numbers > 8 was used. Poly-A RNA was enriched, and sequencing libraries were prepared using the NEBNext Ultra RNA preparation Kit (New England Biolabs, Ipswich, USA) according to the manufacturer’s protocols. Single read sequencing was conducted on an Illumina NextSeq 500 (Illumina, San Diego, USA) with a length of 75 bps.

### Bioinformatical analyses

All raw sequencing data were investigated for their read quality with FastQC v. 0.11.5 [61] to adjust the adapter trimming correctly for Trimmomatic v. 0.36 [62], which has been used with the following parameters to trim the adapters, primers, and quality: ILLUMINACLIP:./adapters/list.fa:2:30:10:6 LEADING:3 TRAILING:3 SLIDINGWINDOW:4:15 MINLEN:36. Only reads with a Phred quality score of $\ge 30$ were considered to ensure high-quality reads and these have been checked additionally with FastQC [61]. All high-quality reads were mapped to the human reference genome GRCh38.78 (Ensembl) using TopHat v. 2.1.1 [63], which uses Bowtie2 v. 2.2.9 [64]. The required raw read counts per transcript have been counted per ENSEMBL-ID of GRCh38.78 with the combination of SAMtools v. 1.3 [65] and HTSeq-count v. 0.6.1p1 [66]. To get the corresponding gene symbols for the ENSEMBL-IDs, the ENSEMBL-IDs were mapped via the Bioconductor packaged org.Hs.e.g.db v. 3.5.0 [67]. A statistical and differential expression analysis using the Bioconductor package DESeq 2 v. 1.12.4 [68] with a significance level of 0.05 for the Wald test and $log_{2}$ fold change of ± 1.0 was done, using as input the resulting raw expression quantification of the 22 000 transcripts from the previous step.

The downstream analysis comprises differential expression analysis and pathway analysis. The differential expression analysis integrates pathway annotation based on the transcriptional signatures of the mono- and combined drug application. Via a rank–rank matrix of the top 100 regulated genes and the most enriched pathways [69], each drug, as well as drug combination is checked for differences in its effects, which are visualized. For our pathway evaluation, the affected pathway has to pass several thresholds in order to be ranked. These thresholds are, p < 0.05, log2FC + − 1.25, affected genes > 5, and the Top50 pathways are selected for upregulation as well as downregulation. If these thresholds are not passed, the corresponding pathway is not shown in the pathway ranking.

The analysis of combination effects, as well as drug synergy was carried out via ranking the differentially expressed genes regarding their importance. Based on the previously computed rank–rank overlap map, the differences between the mono drug application and drug combinations are calculated. Its results imply up- or downregulation and different regulation between the different drugs as well as their combinations.

The Wnt signaling pathway (WP363), as well as the BCR signaling pathway (WP23) [70], show the involvement of the AKT, BTK, mTOR, and PI3K proteins being affected by the treatment (see Additional file 5: Pathways_components). For our pathway evaluation, the affected pathway has to pass several thresholds in order to be ranked. These thresholds are, p < 0.05, log2FC + − 1.25, affected genes > 5, and the Top50 pathways are selected for upregulation as well as downregulation. If these thresholds are not passed, the corresponding pathway is not shown in the pathway ranking Additional file 6: pw_ranking_Idel.

## Supplementary information

**Additional file 1.** FACS plots. Plots of apoptotic (Annexin V FITC+ and Propidium iodide-) and necrotic cells (Annexin V FITC+ and Propidium iodide+) detected by flow cytometry analysis at 72h of pro-B ALL cell lines RS4;11 and SEM exposed with AraC, DEX and IDEL and two drugs combined (AraC+IDEL, DEX+IDEL).

**Additional file 2.** supplement tables. Comparison of the differential expression analysis of RS4;11 and SEM exposed with AraC, DEX and IDEL and two drugs combined (AraC+IDEL, DEX+IDEL) for 72 h.

**Additional file 3.** supplement figures. Heatmaps of the top-100 up- and downregulated genes of the biological triplicates.

**Additional file 4.** xls-file. Additional data of the top-50 regulated pathways and top-20 regulated genes, as well as comparison to existing reference data.

**Additional file 5.** pathway_components. Abstract of the WNT and BCR signaling pathway and the components.

**Additional file 6.** pw_ranking_Idel. Pathway ranking of pathways with components mTOR, BTK, AKT, and PI3K.

## Data Availability

The datasets used and analysed during the current study are available from the corresponding author on reasonable request.
