# Supplementary material for: Combination of the PI3K inhibitor Idelalisib with the conventional cytostatics cytarabine and dexamethasone leads to changes in pathway activation that induce anti-proliferative effects in B lymphoblastic leukaemia cell lines
Source: Cancer Cell Int. 2020 Aug 12;20:390. doi: 10.1186/s12935-020-01431-4 (PMC7425054; doi:10.1186/s12935-020-01431-4)
Supplement: Supplementary file 1 — Additional file 1. FACS plots. Plots of apoptotic (Annexin V FITC+ and Propidium iodide-) and necrotic cells (Annexin V FITC+ and Propidium iodide+) detected by flow cytometry analysis at 72h of pro-B ALL cell lines RS4;11 and SEM exposed with AraC, DEX and IDEL and two drugs combined (AraC+IDEL, DEX+IDEL). [file 12935_2020_1431_MOESM1_ESM.pptx]

## Slide 1
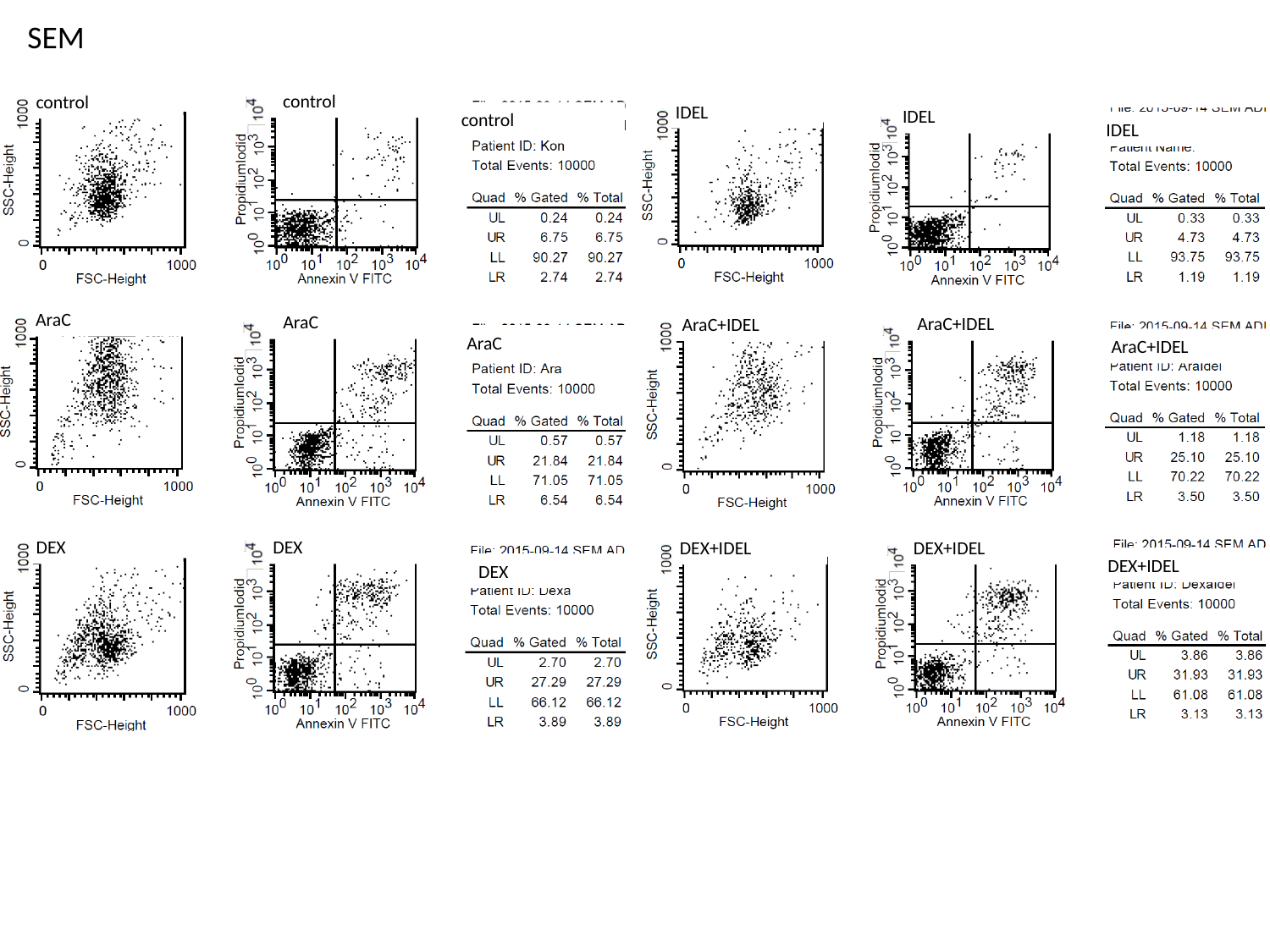

SEM
 control
control
control
IDEL
 IDEL
IDEL
AraC
 AraC
 AraC+IDEL
AraC+IDEL
AraC
AraC+IDEL
DEX
 DEX
DEX+IDEL
 DEX+IDEL
DEX+IDEL
DEX

## Slide 2
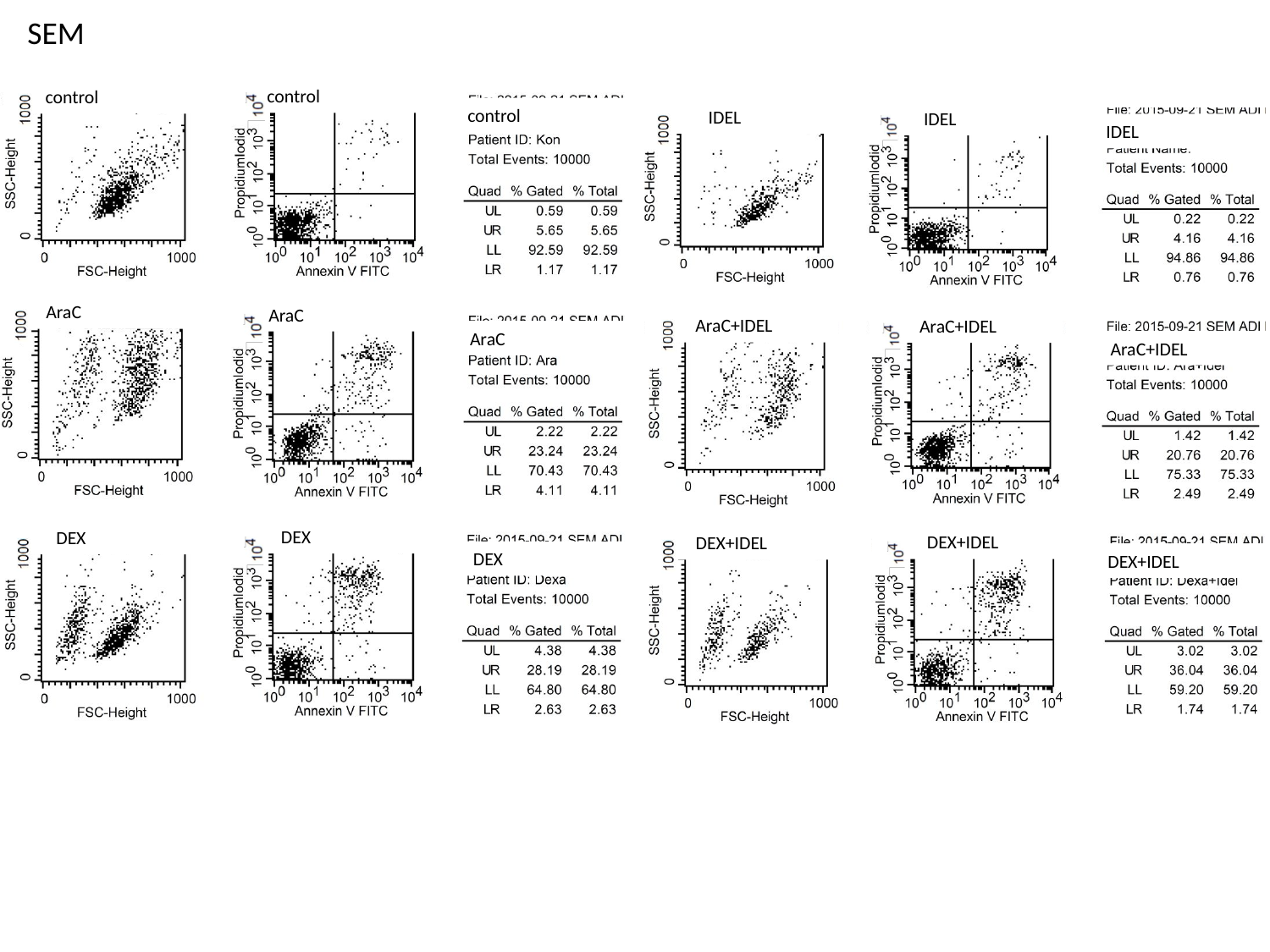

SEM
 control
 control
control
 IDEL
 IDEL
IDEL
 AraC
 AraC
AraC
 AraC+IDEL
 AraC+IDEL
AraC+IDEL
 DEX
 DEX
 DEX+IDEL
 DEX+IDEL
DEX
DEX+IDEL

## Slide 3
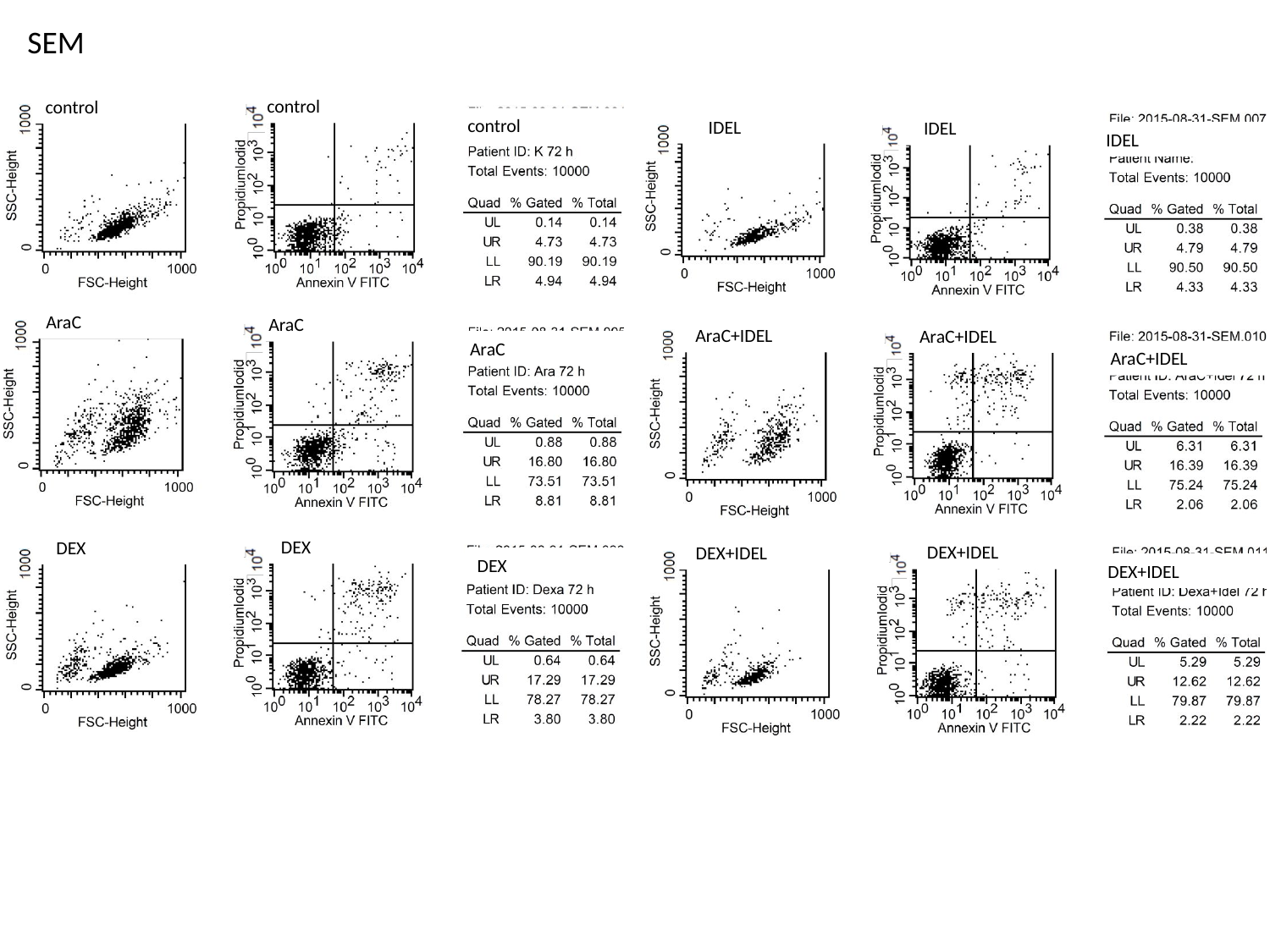

SEM
 control
 control
control
 IDEL
 IDEL
IDEL
 AraC
 AraC
AraC
 AraC+IDEL
 AraC+IDEL
AraC+IDEL
 DEX
 DEX
DEX
 DEX+IDEL
 DEX+IDEL
DEX+IDEL

## Slide 4
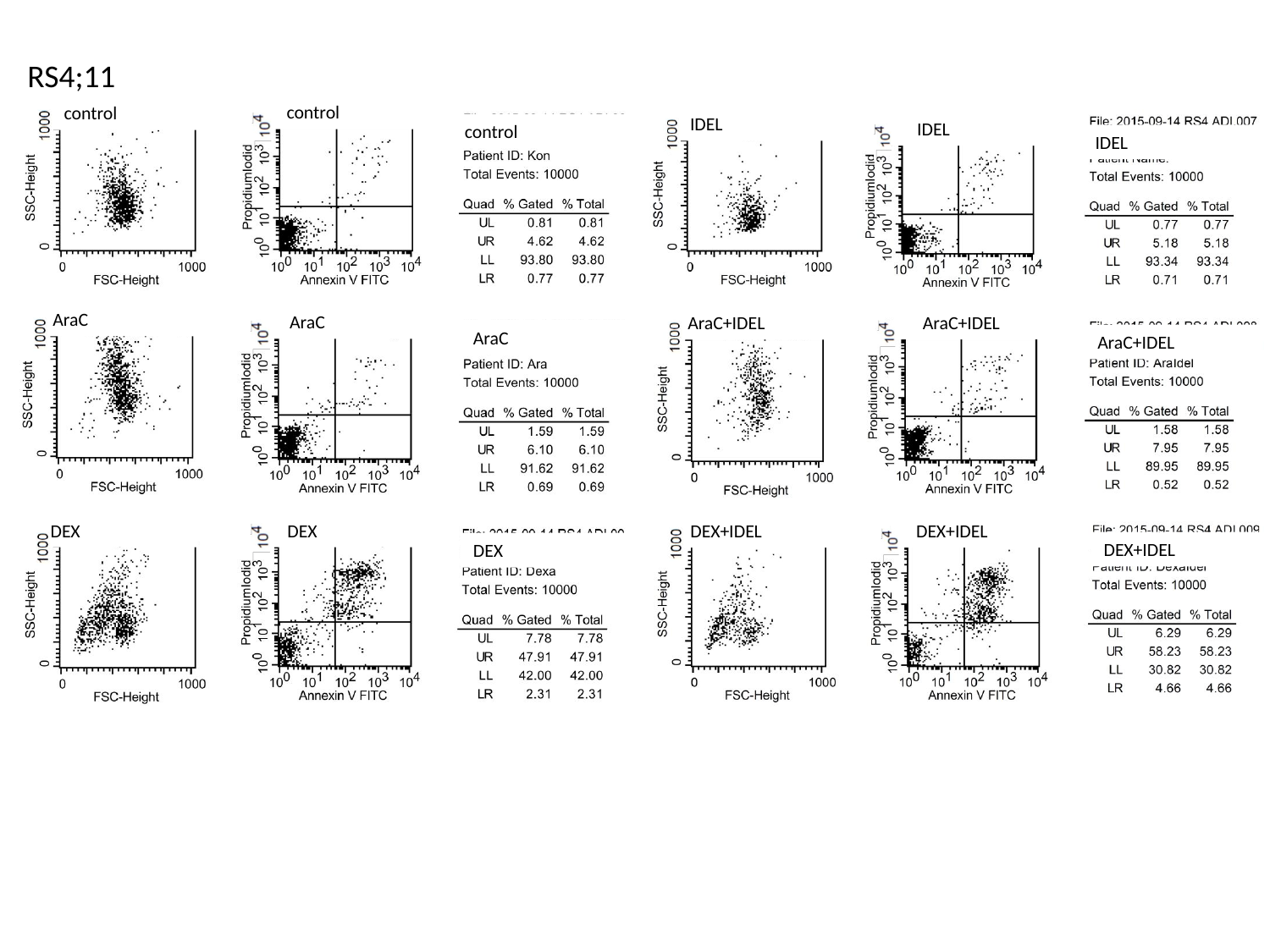

RS4;11
 control
control
control
IDEL
 IDEL
IDEL
AraC
AraC
 AraC
 AraC+IDEL
AraC+IDEL
AraC+IDEL
DEX
 DEX
DEX+IDEL
 DEX+IDEL
DEX+IDEL
DEX

## Slide 5
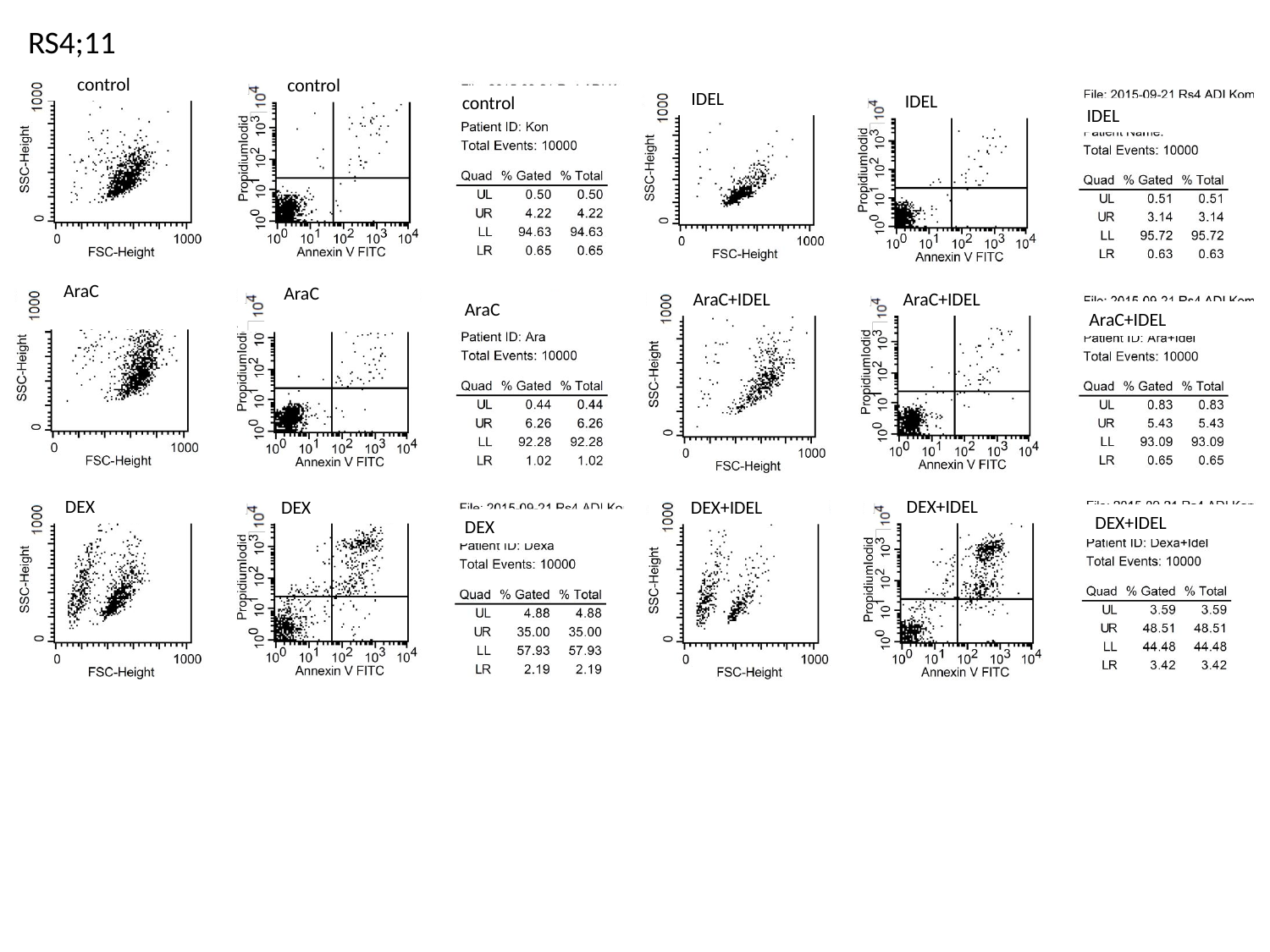

RS4;11
 control
 control
control
 IDEL
 IDEL
IDEL
 AraC
AraC
 AraC
 AraC+IDEL
 AraC+IDEL
AraC+IDEL
 DEX+IDEL
 DEX
 DEX
 DEX+IDEL
DEX+IDEL
DEX

## Slide 6
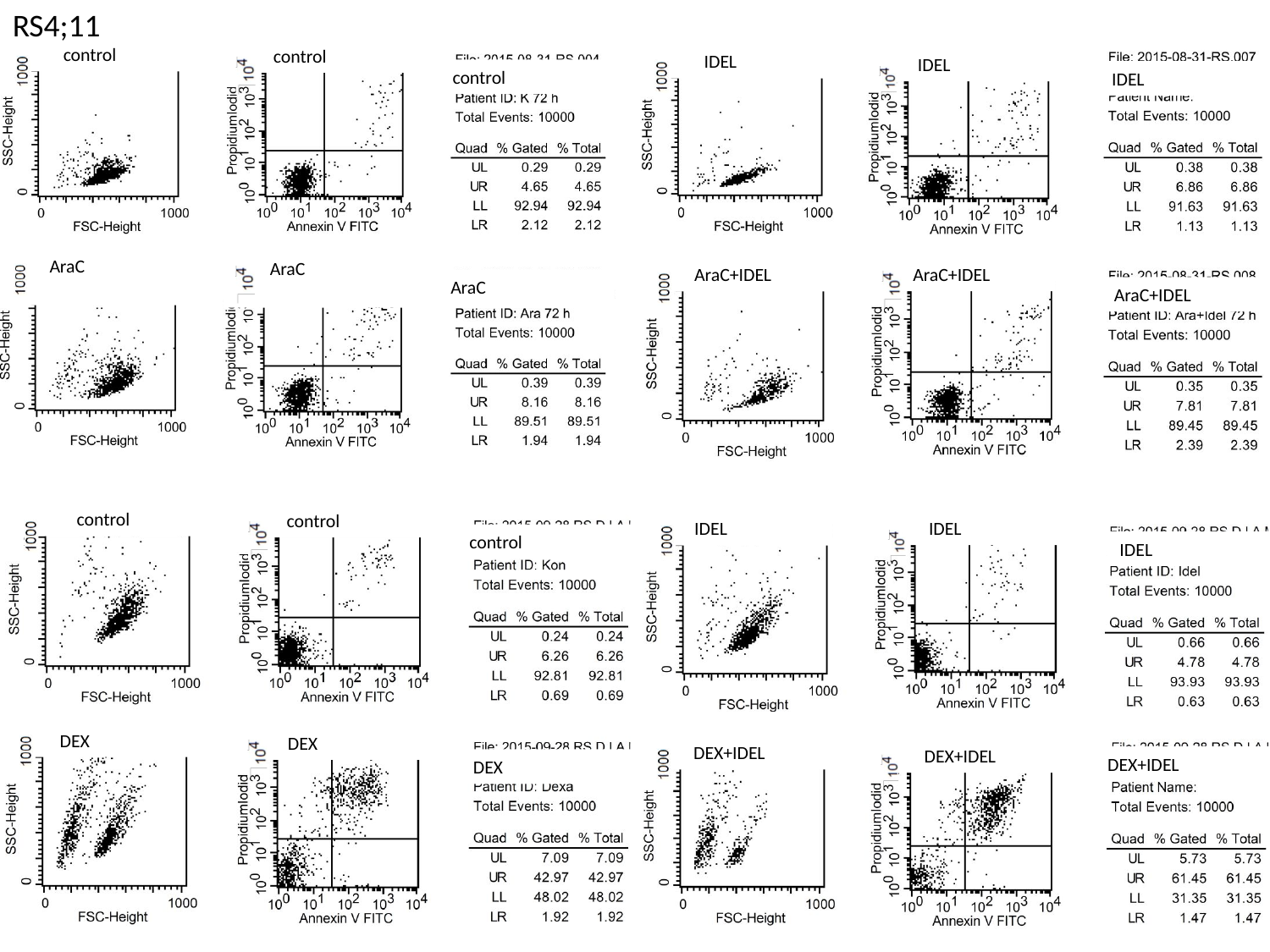

RS4;11
 control
 control
control
 IDEL
 IDEL
IDEL
 AraC
 AraC
AraC
 AraC+IDEL
 AraC+IDEL
AraC+IDEL
 control
 control
control
 IDEL
 IDEL
IDEL
 DEX
 DEX
DEX+IDEL
 DEX+IDEL
DEX
 DEX+IDEL
