# Supplementary material for: Combination of the PI3K inhibitor Idelalisib with the conventional cytostatics cytarabine and dexamethasone leads to changes in pathway activation that induce anti-proliferative effects in B lymphoblastic leukaemia cell lines
Source: Cancer Cell Int. 2020 Aug 12;20:390. doi: 10.1186/s12935-020-01431-4 (PMC7425054; doi:10.1186/s12935-020-01431-4)
Supplement: Supplementary file 2 — Additional file 2. supplement tables. Comparison of the differential expression analysis of RS4;11 and SEM exposed with AraC, DEX and IDEL and two drugs combined (AraC+IDEL, DEX+IDEL) for 72 h. [file 12935_2020_1431_MOESM2_ESM.pptx]

## Slide 1
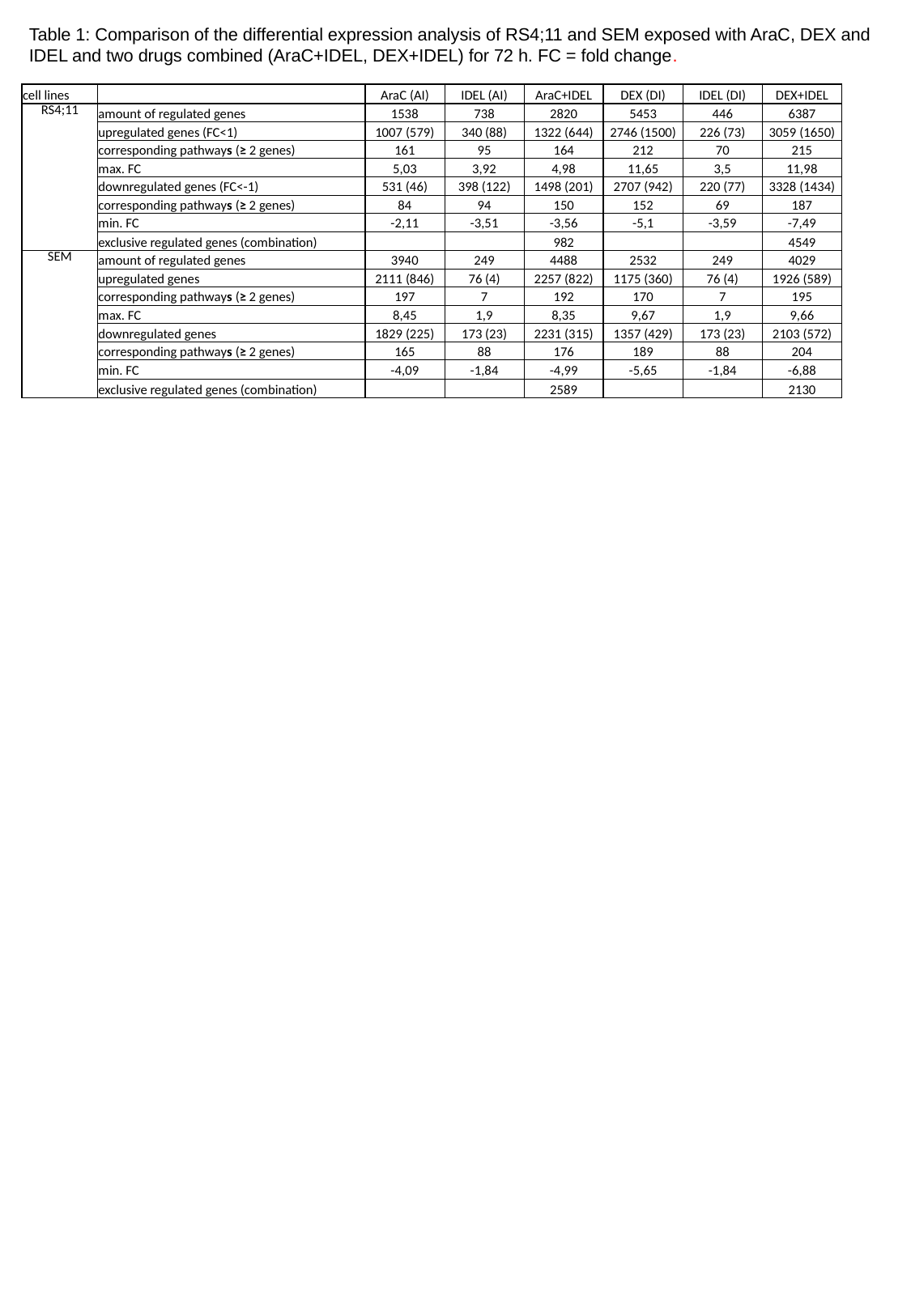

Table 1: Comparison of the differential expression analysis of RS4;11 and SEM exposed with AraC, DEX and IDEL and two drugs combined (AraC+IDEL, DEX+IDEL) for 72 h. FC = fold change.
| cell lines | | AraC (AI) | IDEL (AI) | AraC+IDEL | DEX (DI) | IDEL (DI) | DEX+IDEL |
| --- | --- | --- | --- | --- | --- | --- | --- |
| RS4;11 | amount of regulated genes | 1538 | 738 | 2820 | 5453 | 446 | 6387 |
| | upregulated genes (FC<1) | 1007 (579) | 340 (88) | 1322 (644) | 2746 (1500) | 226 (73) | 3059 (1650) |
| | corresponding pathways (≥ 2 genes) | 161 | 95 | 164 | 212 | 70 | 215 |
| | max. FC | 5,03 | 3,92 | 4,98 | 11,65 | 3,5 | 11,98 |
| | downregulated genes (FC<-1) | 531 (46) | 398 (122) | 1498 (201) | 2707 (942) | 220 (77) | 3328 (1434) |
| | corresponding pathways (≥ 2 genes) | 84 | 94 | 150 | 152 | 69 | 187 |
| | min. FC | -2,11 | -3,51 | -3,56 | -5,1 | -3,59 | -7,49 |
| | exclusive regulated genes (combination) | | | 982 | | | 4549 |
| SEM | amount of regulated genes | 3940 | 249 | 4488 | 2532 | 249 | 4029 |
| | upregulated genes | 2111 (846) | 76 (4) | 2257 (822) | 1175 (360) | 76 (4) | 1926 (589) |
| | corresponding pathways (≥ 2 genes) | 197 | 7 | 192 | 170 | 7 | 195 |
| | max. FC | 8,45 | 1,9 | 8,35 | 9,67 | 1,9 | 9,66 |
| | downregulated genes | 1829 (225) | 173 (23) | 2231 (315) | 1357 (429) | 173 (23) | 2103 (572) |
| | corresponding pathways (≥ 2 genes) | 165 | 88 | 176 | 189 | 88 | 204 |
| | min. FC | -4,09 | -1,84 | -4,99 | -5,65 | -1,84 | -6,88 |
| | exclusive regulated genes (combination) | | | 2589 | | | 2130 |
