# Supplementary material for: Combination of the PI3K inhibitor Idelalisib with the conventional cytostatics cytarabine and dexamethasone leads to changes in pathway activation that induce anti-proliferative effects in B lymphoblastic leukaemia cell lines
Source: Cancer Cell Int. 2020 Aug 12;20:390. doi: 10.1186/s12935-020-01431-4 (PMC7425054; doi:10.1186/s12935-020-01431-4)
Supplement: Supplementary file 3 — Additional file 3. supplement figures. Heatmaps of the top-100 up- and downregulated genes of the biological triplicates. [file 12935_2020_1431_MOESM3_ESM.pptx]

## Slide 1
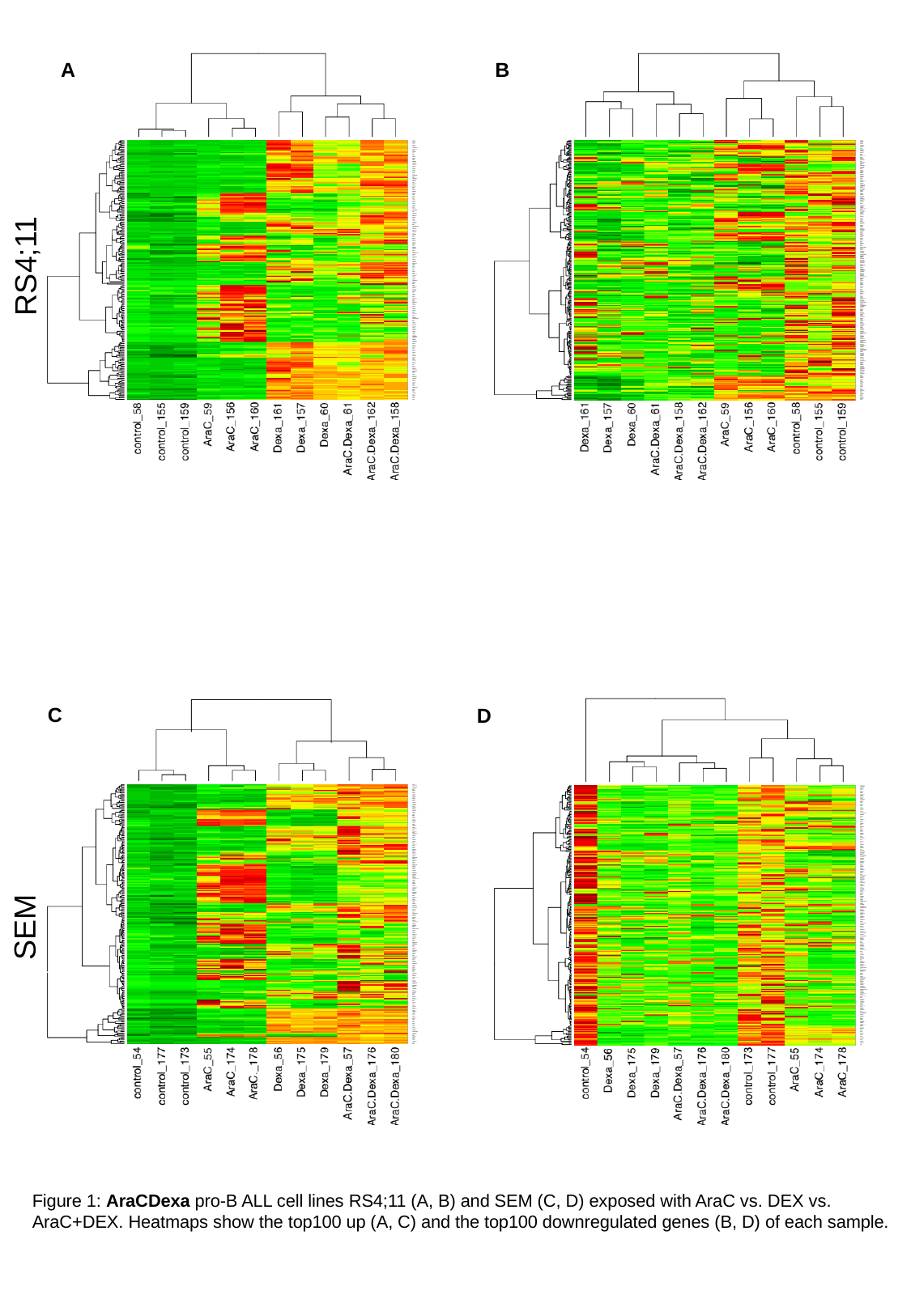

B
A
RS4;11
C
D
SEM
Figure 1: AraCDexa pro-B ALL cell lines RS4;11 (A, B) and SEM (C, D) exposed with AraC vs. DEX vs. AraC+DEX. Heatmaps show the top100 up (A, C) and the top100 downregulated genes (B, D) of each sample.

## Slide 2
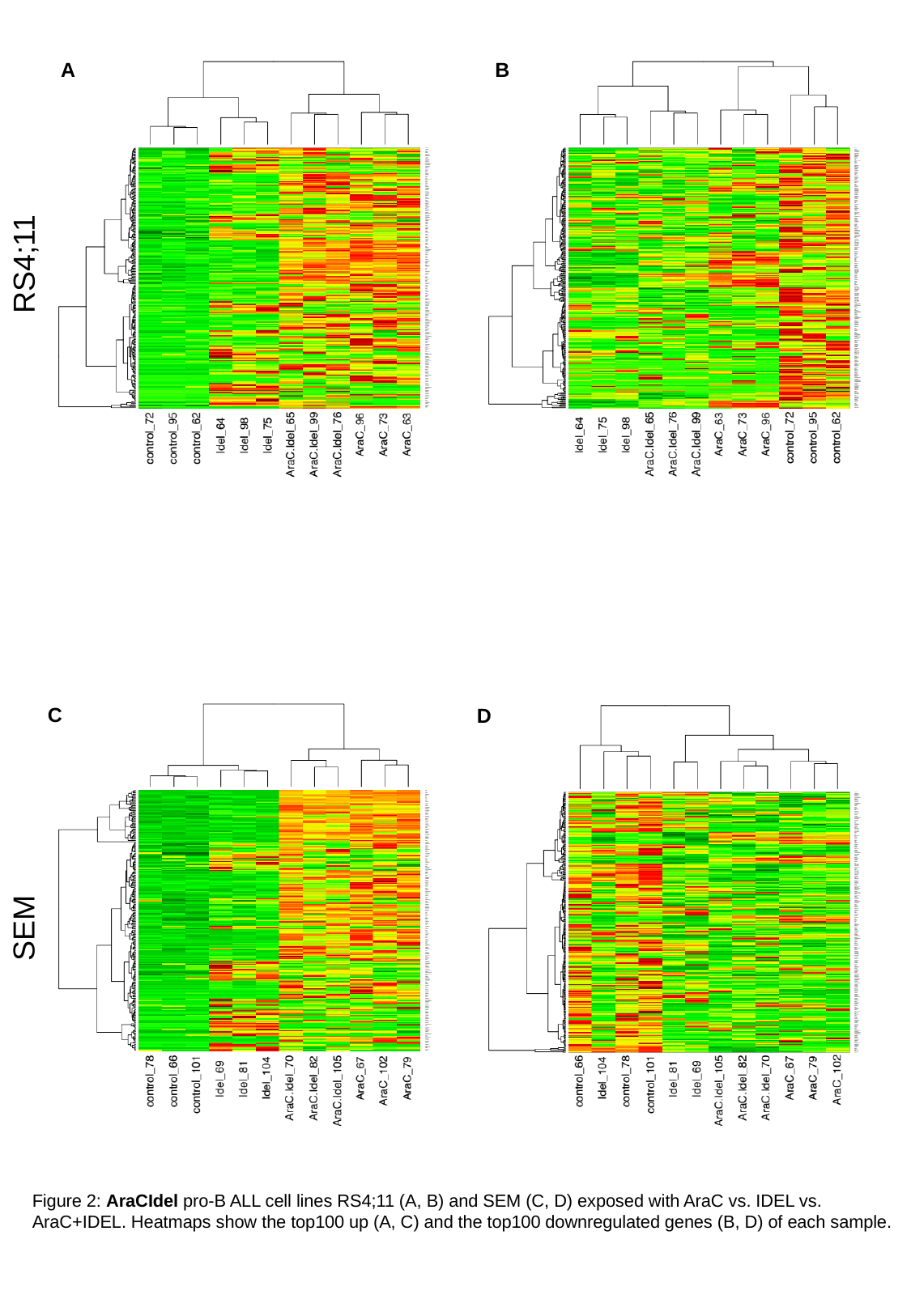

B
A
RS4;11
C
D
SEM
Figure 2: AraCIdel pro-B ALL cell lines RS4;11 (A, B) and SEM (C, D) exposed with AraC vs. IDEL vs. AraC+IDEL. Heatmaps show the top100 up (A, C) and the top100 downregulated genes (B, D) of each sample.

## Slide 3
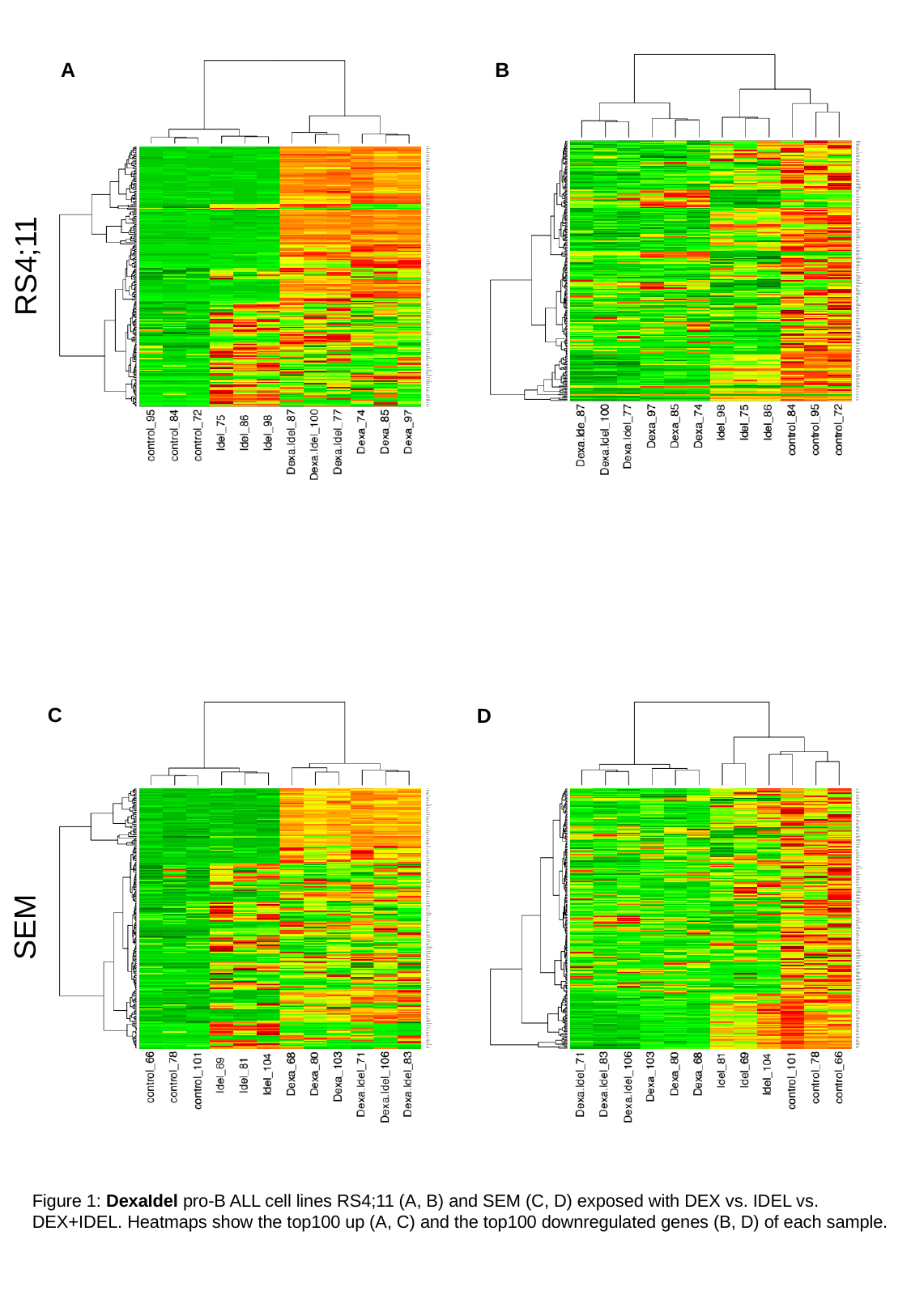

B
A
RS4;11
C
D
SEM
Figure 1: DexaIdel pro-B ALL cell lines RS4;11 (A, B) and SEM (C, D) exposed with DEX vs. IDEL vs. DEX+IDEL. Heatmaps show the top100 up (A, C) and the top100 downregulated genes (B, D) of each sample.
