# Supplementary material for: Combination of the PI3K inhibitor Idelalisib with the conventional cytostatics cytarabine and dexamethasone leads to changes in pathway activation that induce anti-proliferative effects in B lymphoblastic leukaemia cell lines
Source: Cancer Cell Int. 2020 Aug 12;20:390. doi: 10.1186/s12935-020-01431-4 (PMC7425054; doi:10.1186/s12935-020-01431-4)
Supplement: Supplementary file 5 — Additional file 5. pathway_components. Abstract of the WNT and BCR signaling pathway and the components. [file 12935_2020_1431_MOESM5_ESM.docx]

| **Name** | **Type** |
| --- | --- |
| AKT1 | Protein |
| MTOR | Protein |

WNT signaling pathway: WP363 (wikipathways) – not all genes of the 51!!!

***Table 1* Abstract of the WNT signaling pathway (WP363).** The abstract of the in total 51 genes, shows that AKT and mTOR are part of this pathway.

BCR signaling pathway: WP23 (wikipathways) – not all genes of the 103!!!

***Table 2* Abstract of the BCR signaling pathway (WP23).** The abstract of the in total 103 genes, shows that AKT, BTK, and PI3K are part of this pathway.

| **Name** | **Type** |
| --- | --- |
| AKT1 | Protein |
| BTK | Protein |
| PIK3AP1 | Protein |
| PIK3CG | Protein |
| PIK3R1 | Protein |
| PIK3R2 | Protein |
