# Supplementary material for: Combination of the PI3K inhibitor Idelalisib with the conventional cytostatics cytarabine and dexamethasone leads to changes in pathway activation that induce anti-proliferative effects in B lymphoblastic leukaemia cell lines
Source: Cancer Cell Int. 2020 Aug 12;20:390. doi: 10.1186/s12935-020-01431-4 (PMC7425054; doi:10.1186/s12935-020-01431-4)
Supplement: Supplementary file 6 — Additional file 6. pw_ranking_Idel. Pathway ranking of pathways with components mTOR, BTK, AKT, and PI3K. [file 12935_2020_1431_MOESM6_ESM.docx]

| **RS4;11** | | **Treatment** | **SEM** | |
| --- | --- | --- | --- | --- |
| BCR | √ (31 up 36 down) | AD | √ (33 up, 7 down) | BCR |
| WNT | √ (43 up) |  | √ (35) | WNT |
| BCR | √ (11 up) | AI | √ (2 up, 36 down) | BCR |
| WNT | - |  | √ (34 down) | WNT |
| BCR | √ (20 up) | DI | √ (2 up) | BCR |
| WNT | √ (25 up) |  | √ (35 down) | WNT |

In combination!

***Table 1* Pathway ranking of pathways with mTOR, BTK, AKT, and PI3K of the combination treatment.** For each combination treatment (AD, AI, DI), the pathway ranking position of up- and downregulation are shown for the BCR signaling and WNT signaling pathways. The row is empty, in case the thresholds are not passed.

Monotreatment

| **RS4;11** | | **Treatment** | **SEM** | |
| --- | --- | --- | --- | --- |
| BCR | √ (9 up) | Idel  (AI) | √ (1 up) | BCR |
| WNT | √ (42 up) |  | √ (50 down) | WNT |
| BCR | √ (24 up) | Idel  (DI) | √ (1 up) | BCR |
| WNT | - |  | √ (down) | WNT |

***Table 2* Pathway ranking of pathways with mTOR, BTK, AKT, and PI3K of Idelalisib.** For the Idelalisib treatment, the pathway ranking position of up- and downregulation are shown for the BCR signaling and WNT signaling pathways. The row is empty, in case the thresholds are not passed.
